# STEP_61_ is a regulator of mGluR5 expression

**DOI:** 10.1016/j.neuropharm.2026.111198

**Published:** 2026-09-28

**Authors:** Sehoon Won, Kai Chang, Xiaobing Chen, Katherine W. Roche

**Affiliations:** a Receptor Biology Section, National Institute of Neurological Disorders and Stroke, National Institutes of Health, Bethesda, MD, 20892, USA; b Laboratory of Neurobiology, National Institute of Neurological Disorders and Stroke, National Institutes of Health, Bethesda, MD, 20892, USA

**Keywords:** mGluR5, STEP_61_, Tyrosine dephosphorylation, Surface expression, Electron microscopy

## Abstract

Metabotropic glutamate receptor 5 (mGluR5) is a Gq protein-coupled receptor that modulates both excitatory and inhibitory neurotransmission and regulates numerous higher-order neural processes ranging from learning and memory to mood and behavior. Trafficking of mGluR5 to the plasma membrane is critical for its biological functions and phosphorylation is an important regulator of mGluR5 trafficking and function. We previously reported that striatal-enriched protein tyrosine phosphatase 61 (STEP_61_) interacts with mGluR5 in a proteomics study of the STEP_61_ interactome. Here, we explored the relationship between STEP_61_ and mGluR5 in greater detail and find that STEP_61_, like mGluR5, is preferentially localized to extra-synaptic sites in neurons using electron microscopy. A systematic evaluation of STEP_61_ in heterologous cells identifies its kinase-interacting and amino-terminal domains as being important for mGluR5 binding. Interestingly, we find that STEP_61_ decreases mGluR5 surface and total protein levels in both heterologous cells and neurons in a lysosome-dependent manner. Conversely, mGluR5 protein and tyrosine phosphorylation levels are increased in STEP knockout (KO) mouse brain. There are four tyrosine residues in the cytosolic tail region of mGluR5, and their mutation decreases total and surface receptor protein levels. Together, these findings reveal an important role of STEP_61_-mediated tyrosine dephosphorylation as a negative regulator of mGluR5 surface protein levels and stability.

## 1. Introduction

mGluRs (mGluR1–8) are class C G-protein coupled receptors (GPCRs) expressed throughout the central nervous system and are important for synaptic transmission and plasticity (Niswender and Conn, 2010; Nakanishi, 1994; Suh et al., 2018; Vaidya et al., 2013). Group I mGluRs (mGluR1 and mGluR5) are expressed in distinct neuronal populations where they couple receptor binding to phospholipase C (PLC) activation via Gq/_11_, resulting in intracellular Ca^2+^ increase and protein kinase C (PKC) stimulation (Ribeiro et al., 2010; Suh et al., 2018). Unlike group II (mGluR2/3) and group III (mGluR4/6–8) mGluRs, group I mGluRs (1/5) are primarily located post-synaptically (Bodzeta et al., 2021). mGluR5 is highly enriched in hippocampus and cortex where it regulates neuronal excitability and modulates the strength of LTP and LTD (Niswender and Conn, 2010; Heidinger et al., 2002). Furthermore, mGluR5 regulates the expression and function of ionotropic glutamate receptors, including the N-methyl-D-aspartate (NMDA) receptor subunits GluN1 and GluN2B (Heidinger et al., 2002; Suh et al., 2018). Aberrant mGluR5 expression and signaling have been reported in clinical studies of various neurological disorders such as anxiety, depression, addiction, autism and Alzheimer’s disease (Vaidya et al., 2013), as well as lymphoma (Schnell et al., 2023) and melanoma (Choi et al., 2011a,b). Many negative and positive allosteric modulators (NAMs and PAMs) have been developed to target mGluR5 (Niswender and Conn, 2010; Vaidya et al., 2013). mGluR5 antagonists have been shown to have pharmacological effects in reducing synaptic plasticity, altering NMDA receptor function, and protecting against excitotoxicity and neuroinflammation. Expression of mGluR5 can be altered in neurological and psychiatric conditions. In addition, mGluR5 expression has been used as a biomarker to monitor disease progression and treatment response in Alzheimer’s disease (AD) (Abd-Elrahman et al., 2020; Wang et al., 2024). Therefore, studies on the regulation of mGluR5 expression are of interest to basic neuroscientists as well as for drug development.

Tyrosine phosphorylation regulates both ionotropic and metabotropic GluRs (Mao and Wang, 2016). The balance between tyrosine phosphorylation and dephosphorylation is an important mechanism for maintaining cellular homeostasis and requires precise regulation of various tyrosine kinases and phosphatases (Lemmon and Schlessinger, 2010). Tyrosine phosphorylation in receptor tyrosine kinases (RTKs) is regarded as a pivotal step for cell proliferation, differentiation and apoptosis (Lemmon and Schlessinger, 2010), while phosphorylated tyrosine in ionotropic and metabotropic glutamate receptors is crucial for receptor activation, downstream signaling, and trafficking. mGluR5 is the most abundant subtype among eight mGluRs located in the striatum where both tyrosine kinases and phosphatases are also enriched. mGluR5 contains four tyrosine residues located in its long C-tail, and their phosphorylation states have been found to be responsible for not only its own functionality and but also that of other glutamate receptors, leading to changes in synaptic strength, plasticity and homeostasis (Heidinger et al., 2002). The role of mGluR5 posttranslational modifications in regulating trafficking has been investigated in terms of serine and threonine phosphorylation, whereas little is known about tyrosine phosphorylation (Choi et al., 2011a,b; Kim et al., 2008; Ko et al., 2012; Lee et al., 2008).

Striatal-enriched protein tyrosine phosphatase [STEP, also known as protein tyrosine phosphatase nonreceptor type 5 (PTPN5)] is the only brain-specific tyrosine phosphatase that is expressed in cortex, hippocampus, and striatum (Boulanger et al., 1995; Bult et al., 1996; Johnson and Lombroso, 2012; Snyder et al., 2005). There are several splice variants (STEP_61_, STEP_46_, STEP_38_, and STEP_20_), all of which are expressed in the striatum whereas only STEP_61_ is expressed in cortex and hippocampus (Bult et al., 1997; Johnson and Lombroso, 2012; Won et al., 2016). STEP_61_ is a multi-domain protein consisting of two proline rich domains and two transmembrane regions at the amino-terminus (N-terminus), kinase-interacting motif (KIM) and kinase-specificity sequence (KIS) domains in the center, and a protein tyrosine phosphatase domain (PTP) at the carboxyl terminus (C-terminus) (Bult et al., 1997; Jang et al., 2015; Won et al., 2019). Previous studies have shown that STEP_61_ binds to ionotropic GluRs including both NMDA receptors and AMPA receptors and regulates their synaptic or extra-synaptic trafficking by dephosphorylating tyrosine residues located in their intracellular tail regions (Jang et al., 2015; Snyder et al., 2005; Won et al., 2019). STEP dysfunction has been implicated in neurological and neuropsychiatric disorders (Braithwaite et al., 2008; Carty et al., 2012; Goebel-Goody et al., 2012; Johnson and Lombroso, 2012; Saavedra et al., 2011).

We previously reported mGluR5 as a STEP_61_ binding protein in a proteomics study of the STEP_61_ interactome (Won et al., 2019). In this study, we show that STEP_61_ is enriched at extra-synaptic sites in cultured primary neurons, similar to mGluR5 (Hubert et al., 2001; Lujan et al., 1996; Negyessy et al., 1997). We characterized STEP_61_ binding to mGluR5 in brain tissue and primary neurons and map the protein domains mediating its interaction with mGluR5. Interestingly, STEP_61_ expression greatly decreases mGluR5 surface trafficking in heterologous cells and primary neurons and strongly reduces mGluR5 protein levels through the lysosomal degradation pathway. Conversely, mGluR5 protein and tyrosine phosphorylation levels are significantly increased in synaptic plasma membrane (SPM) fractions of adult STEP KO brains. Consistent with a critical role of tyrosine phosphorylation, mutating all four tyrosine residues in the intracellular C-terminal tail reduces mGluR5 surface expression and total protein expression. Together, our findings reveal an important role of STEP_61_-mediated tyrosine dephosphorylation in regulating mGluR5 surface expression and protein stability.

## 2. Materials and methods

### 2.1. Plasmid DNAs and antibodies

HA- and myc-tagged rat mGluR5 expression constructs in the pRK5 or pcDNA3.1 vector were described previously (Choi et al., 2011a,b). The myc-tagged mGluR5 plasmid was used as template for mutagenesis using the QuikChange Site-Directed Mutagenesis kit (Agilent) and a modified protocol (Chang and Roche, 2017). Tyrosine phosphorylation-deficient mutants, Y821A, Y963A, Y1060A, Y1155A, Y1163A and 4 YAs (Y963A/Y1060A/Y1155A/Y1163A), were also made in the myc-mGluR5 plasmid. HA- or myc-tagged STEP_61_ or STEP_46_, various truncated forms of STEP_61_ (Won et al., 2016, 2019) and constitutively active Fyn constructs were previously published (Prybylowski et al., 2005). All truncations and mutations of mGluR5 and STEP cDNA were all confirmed by DNA sequencing. PSD-95-EYFP was generated with EYFP fused in frame at the C-terminal end of rat PSD-95 in pCAGGS vector (Chen et al., 2011). The following commercial antibodies were used: Mouse anti-myc (9B11), rabbit anti-myc (71D10), mouse anti-HA (6E2), and rabbit anti-HA (C29F4) from Cell Signaling Technology (Danvers, MA); mouse anti-phosphotyrosine (4G10) and rabbit anti-mGluR5 (AB5675) from Millipore; rabbit anti-mGluR1 (12551) from Cell Signaling; mouse anti-STEP (23E5) from Novus Biologicals; mouse anti-GFP (N86/38) from Neuromab; mouse anti-actin (G043) from Applied Biological Materials; and mouse anti-GAPDH (G-9) from Santa Cruz Biotechnology. Fluorophore-conjugated secondary antibodies were from Thermo Fisher Scientific (Invitrogen, Grand Island, NY), and peroxidase labeled secondary antibodies were from Cytiva.

### 2.2. Animals

All animal procedures were approved by the National Institute of Neurological Disorders and Stroke (NINDS) Animal Care and Use Committee (protocol #1171) and performed in accordance with established NIH Animal Care and Use of Laboratory Animal guidelines. C57BL/6 and STEP KO mice, and Sprague-Dawley rats were housed in a specific pathogen-free and well-ventilated animal facility at NINDS. Mice and rats were housed in a temperature and humidity-controlled holding rooms under a 12 h light/dark cycle and provided supportive care (i.e. standard diet, drinking water, and diet gel) by animal facility staffs. Timed pregnant rats (Charles River laboratories) were received at least 1 day prior to experiment. All animals were euthanized in a carbon dioxide (CO_2_) chamber. Every possible effort and precaution were taken to minimize pain to the animals throughout the experimental procedures.

### 2.3. Co-immunoprecipitation (co-IP) of endogenous STEP_61_ in mouse brain tissue and rat primary cultured neurons

Synaptosomes from adult mouse forebrain and rat primary cultured neurons at days *in vitro* (*DIV*) 28 were solubilized with 1% Doc lysis buffer [1% sodium deoxycholate, 50 mM Tris-HCl (pH 8.8), 1 mM EDTA, Complete^®^ protease inhibitor cocktail (Roche, 11697498001), and phosphatase inhibitor cocktails II (Sigma, catalog no. P5726) and III (Sigma, catalog no. P0044)] for 30 min at 37 °C, then neutralized with 1% Triton X-100 lysis buffer [1% Triton X-100, 50 mM Tris-HCl (pH 7.5), 1 mM EDTA, 150 mM NaCl_2_, protease inhibitor cocktail, and phosphatase inhibitor cocktails II and III] for 30 min on ice. The lysate was centrifuged at 100,000 × g for 20 min at 4 °C and the resultant supernatant was used for co-IP. 1 μg anti-STEP antibody was added into the lysate and incubated overnight at 4 °C. The next day, 15~20 μl of Protein G-Sepharose beads were added and incubated for 3~4 h at 4 °C. Following 3 washes with 1% Triton X-100 lysis buffer, 30 μl of 2X SDS sample buffer was added to the beads for elution and incubated for 20 min at 42 °C.

### 2.4. Co-immunoprecipitation of overexpressed mGluR5 and STEP_61_ in HEK293T cell and immunoblotting

HEK293T cells grown in 35 or 60 mm dishes were co-transfected with HA-STEP_61_ and truncated myc-mGluR5 or HA-mGluR5 and truncated myc-STEP_61_ for 24~48 h. After three washes with cold PBS, transfected HEK293T cells were collected, and cell pellets were resuspended with 50 μl PBS and immediately lysed with 0.5 ml of 1% Triton X-100 lysis buffer or RIPA buffer. Cell lysates were then incubated for 60 min at 4 °C with rocking, and soluble lysates were cleared by microcentrifugation for 30 min at 4 °C. 2~4% of lysates were saved and each lysate was incubated with 2 μg of rabbit anti-HA (for IP STEP) or 1 μg of rabbit anti-mGluR5 or 1 μg of mouse pan-phospho-tyrosine (4G10) antibody at 4 °C overnight. 20 μl of Protein A-Sepharose beads (for rabbit antibody) or Protein G beads (for mouse antibody) was then added and incubated at 4 °C for 3~4 h. After three washes with lysis buffer and centrifugation at 7,000 rpm for 1 min, 30 μl of 2X SDS sample buffer was added to the beads and the beads were incubated at 42 °C for 20 min before loading and resolving on a 7~8% gel for SDS-PAGE. After the gel transfer to PVDF membrane, the blot was cut off at 100 kDa molecular weight line and was probed with mouse anti-myc (for mGluR5), rabbit anti-mGluR5, mouse anti-HA (for STEP proteins), and mouse anti-STEP overnight at 4 °C. After 6 washes with TBS/0.5% Tween 20 (TBST), 1:5000 diluted secondary HRP-conjugated anti-mouse antibodies were incubated at room temperature (RT) for 1~2 h, followed by 30~60 min washes in TBST and ECL development.

### 2.5. Transfection and immunofluorescence staining

Immunofluorescence (IF) staining and microscopy were performed on cultured hippocampal neurons or HEK293T cells and were performed independently at least 3 to 5 times. Rat hippocampal neurons were plated onto poly-D-lysine (PDL)-coated round glass 18 mm coverslips in 12-well plates with 2 ml neurobasal (NB) media containing B-27 supplement and GlutaMAX. Half of the media was replaced on *DIV* 7 with NB media only, and the neuronal cultures were transfected on *DIV* 10 or 11 with 1~2 μg of plasmid DNA and 1~2 μl of Lipofectamine (2000) in 200 μl NB media added to each well containing 1 ml conditioned culture media and incubated overnight. 1 ml of pre-warmed NB media was then added back to each well and the neurons were processed for IF on *DIV* 12 or 13. HEK293T cells were plated onto PDL-coated 18 mm coverslips in a 12-well plate at 1 ml/well were transfected with 1~2 μg of plasmid DNA and 3 μl of polyethylenimine (1 mg/ml in H_2_O, pH 7.0) in 100 μl of OptiMEM (Thermo Fisher) for 16~20 h, followed by IF. Double or triple IF staining for cell surface and intracellular myc-mGluR5 in neurons and heterologous cells was performed similarly except antibody surface labeling in neurons was done at RT, whereas it was done on ice for 30 min in HEK293T cells. After a brief wash in PBS plus calcium, cells were fixed in 4% paraformaldehyde and 4% sucrose in PBS for 10 min, followed by PBS washes and blocking in 10% normal goat serum (NGS) in PBS at RT for 10 min. Cells were then incubated with Alexa Fluor 555 labeled secondary antibodies in 3% NGS in PBS at RT for 30 min, followed by a brief wash in PBS, a 5~10 min permeabilization in 0.25% Triton X-100 in PBS, and an incubation in 10% NGS in PBS. Intracellular labeling was performed with primary antibody and secondary Alexa Fluor 647- or 488-labeled secondary antibodies, both at RT for 30 min and each followed by PBS washes. Coverslips were then mounted on slides using ProLong AntiFade mounting media (Thermo Fisher).

### 2.6. Confocal microscopy and quantification

All IF images were taken using a Zeiss LSM510 confocal microscope with a 40X objective at 512× 512 pixels resolution. Images of 5 to 10 transfected neurons or 5 to 10 focal fields of HEK293T cells were captured from each coverslip. In general, Alexa Fluor 555 was used for surface labeling, Alexa Fluor 647 for intracellular labeling, and Alexa Fluor 488 for intracellular labeling of a co-expressed protein in triple IF experiments. MetaMorph software was used for quantification. 5 to 10 transfected neurons or 5 to 10 image fields of heterologous cells for each experiment were quantified for total intensity for 10 different regions with two or three different fluorescent signals simultaneously. Between 3 and 5 independent experiments were performed for each transfection and all the quantified intensity data were collected for biostatistics analysis. Significance was determined with adjusted *p* value < 0.05 using a one-way ANOVA as presented in a graph.

### 2.7. Cell surface biotinylation assay

Surface biotinylation assay was performed as described previously (Won et al., 2016), and all steps were performed at 4 °C. HEK293T cells were co-transfected with HA-STEP_61_ and myc-mGluR5 or HA-STEP_61_ and myc-mGluR5–4YAs. After 24 h, cells were washed with cold PBS (including 2 mM CaCl_2_ and 1 mM MgCl_2_) and then were incubated with 0.5–1.0 mg/mL biotin (EZ Link Sulfo-NHS-LC-Biotin, Thermo Fisher Scientific) in PBS (containing CaCl_2_ and MgCl_2_) at 4 °C for 30 min. Next, cells were incubated for 15 min with 100 mM glycine in PBS (containing CaCl_2_ and MgCl_2_) to quench unreacted biotin, followed by a PBS wash. Cells were then collected in PBS at 4 °C, and cell pellets were lysed with RIPA buffer [50 mM Tris-HCl (pH 7.4), 1 mM EDTA, 150 mM NaCl, 1% NP-40, 0.5% sodium deoxycholate, 0.1% SDS] at 4 °C for 1 h, followed by centrifugation at 12,000 rpm for 10 min at 4 °C. Supernatants were then incubated with 15~20 μl of Neutravidin agarose beads (Thermo Fisher Scientific) equilibrated in RIPA buffer with agitation for 1~2 h. Following three washes with RIPA buffer, biotinylated proteins were eluted in 2X SDS sample buffer for 20 min at 42 °C, resolved by SDS-PAGE and analyzed by Western blotting.

### 2.8. Purification of synaptosomes and synaptic plasma-membrane (SPM) from mouse brain

Fractionation experiments were performed following standard methods (Hallett et al., 2008; Won et al., 2016, 2019). Brain tissue from WT or STEP-KO mice (purchased from The Jackson Laboratory) was homogenized on ice with cold TES buffer [10 mM Tris-HCl (pH 7.5), 5 mM EDTA, 320 mM sucrose, protease inhibitor mixture, and phosphatase inhibitor mixture II and III]. Homogenates were then centrifuged at 1000 × g for 10 min at 4 °C, and S1 supernatants were centrifuged at 10,000 × g for 20 min at 4 °C to obtain the crude synaptosomal P2 membrane fractions. Crude synaptosomal membranes were resuspended in ice-cold hypotonic TES buffer [10 mM Tris-HCl (pH 7.5), 5 mM EDTA, 35.6 mM sucrose, protease and phosphatase inhibitors] for 30 min, followed by centrifugation at 25,000 × g for 20 min at 4 °C to obtain synaptic plasma membranes (SPMs). SPMs were solubilized with 1% SDS lysis buffer [1% SDS, 50 mM Tris-HCl (pH 7.5), 1 mM EDTA, protease and phosphatase inhibitors] for 30 min at 37 °C, then neutralized with 1% Triton X-100 lysis buffer [1% Triton X-100, 50 mM Tris-HCl (pH 7.5), 1 mM EDTA, 150 mM NaCl_2_, protease and phosphatase inhibitors] for 30 min on ice. Samples were centrifuged at 100,000 × g for 20 min at 4 °C, and supernatants were used for phosphor-tyrosine IP using the 4G10 antibody.

### 2.9. Immunogold EM labeling

The animal protocol for using time pregnant rat to make dissociated hippocampal culture was approved by the National Institute of Neurological Disorders and Stroke/National Institute of Deafness and Communication Disorders/National Center for Complementary and Integrative Health Animal Care and Use Committee (ACUC) and conforms to NIH guidelines (Approval #1159 for Tom Reese).

Dissociated rat hippocampal neurons from embryonic day 20 pups were plated on a confluent layer of glia grown on round 25 mm nitric acid-treated and PDL-coated German glass coverslips (Warner Instruments). Cultures were maintained in 10% CO_2_ at 35 °C in custom media containing MEM supplemented with 5% horse serum, 2% fetal bovine serum, 2 mM Glutamax (Thermo Fisher), 136 μM uridine 54 μM 2-deoxy-5-fluoro-uridine (Sigma), and N3 supplement (Chen et al., 2008, 2011, 2014, 2015).

Three-week old cultures were transfected with in-frame fusion constructs of PSD-95-EYFP, GFP-STEP_61_, myc-STEP_61_ using either calcium phosphate (Jiang and Chen, 2006) or magnetofection method (OZ Science). 2~4 μg of cDNA per culture dish was used in minimal MEM medium, followed by ~20 h incubation in growth medium. Cultures transfected with GFP fusion proteins were first examined on a LSM510 confocal microscope (Zeiss) to confirm adequate transfection efficiency.

For immunogold labeling, 2 independent coverslips with cultured neurons were typically used in parallel for a particular primary antibody. Neurons were fixed in 4% paraformaldehyde in 0.1 M PBS for 40–45 min, then washed 4-time with PBS. Cells were then permeabilized and blocked with 0.1% saponin and 5% normal goat serum (NGS) in PBS for 1 h at the room temperature. Cells were then incubated with primary antibodies diluted in the blocking solution for 1 h at room temperature. Following several washes with 1% NGS and 0.02% saponin in PBS, cells were incubated with a Nanogold-conjugated secondary Fab antibody fragment (Nanoprobes) diluted in 1% NGS and 0.02% saponin for 1 h at the room temperature, then washed in PBS containing 1% NGS and 0.02% saponin. Finally, labeled cells were post-fixed in 2% glutaraldehyde for 1 h at the room temperature and stored in 4 °C in the fixative solution for further EM processing. Nanogold particles were enlarged in a dark room for ~8 min using a silver-enhancement HQ kit (Nanoprobes), then rinsed in 0.1 M cacodylate buffer and treated with 0.2% osmium tetroxide for 30 min on ice followed by 0.25% uranyl acetate overnight. Samples were dehydrated in 50, 70, and 90% EtOH, followed by three 7-min rinses with 100% EtOH. Next, samples were infiltrated with 1:1 Epon/EtOH for 30 min at room temperature, then with 2:1 Epon/EtOH for 30 min at room temperature and finally with 100% Epon for 10 min at 47 °C and twice more for 1 h at 47 °C. Polymerization was done in a 50 °C oven overnight, followed by transfer to a 60 °C oven (Chen et al., 2018). Resin blocks with coverslips were examined under a light microscope to mark labeled neurons. Coverslips were then released from the Epon blocks by brief immersion in liquid nitrogen and marked areas were cut out with a saw and mounted for thin sectioning. For conventional EM morphological analyses, sections were either cut face to a thickness of ~70 nm and grid stained with uranyl acetate and lead citrate, or into 200 – 300 nm thick sections without grid staining.

### 2.10. EM imaging and image analysis

Transmission EM images were acquired either on a JOEL 200 cx or a JOEL-1400 transmission electron microscope equipped with a bottom mounted CCD camera (2624 × 2624 pixels or 6576 × 4384 pixels AMT, Woburn, MA). Distances measured from the center of immunogold to the post-synaptic membrane or to the closest edge of the PSD were performed in Fiji (NIH ImageJ). To minimize the effects of STEP_61_ overexpression, only immunogold particles closest to the plasma membrane were included in the particle-membrane distance measurements. Lateral distance of mGluR5 label to the edge of the PSD was measured in Fiji by first drawing a curve parallel to the post-synaptic membrane from the center of gold particle to the edge of the PSD, then measuring the length of that curve. Quantification and statistical analyses were performed with Prism (GraphPad). Statistical results are reported as mean ± SD. Levels of statistical significance: *p < 0.05; **p < 0.01; ***p < 0.001; ****p < 0.0001.

### 2.11. Quantification and statistical analysis

Densitometric Western blotting data were quantified with ImageJ and integrated intensity values from confocal imaging regions of interest were quantified with MetaMorph software. Data were tabulated and graphed in Excel file or Prism 7. Comparisons between two conditions were tested for statistical significance using Student’s *t-test*, while comparisons between three or more conditions were tested for significance using one-way ANOVA with Dunnett’s post hoc test. *P* values < 0.05 were considered significant.

## 3. Results

### 3.1. STEP_61_ interacts with mGluR5 and, like mGluR5, localizes to extra-synaptic region

We previously reported the STEP_61_ interactome that included many synaptic proteins (Won et al., 2019). To reliably and reproducibly perform co-immunoprecipitation (co-IP) and identify STEP_61_ interacting proteins using in-gel mass spectrophotometry, we modified the standard sample preparation in two important ways: first, to increase sensitivity and exclude proteins that bind to other STEP isoforms (i.g. STEP_46_, STEP_38_, and STEP_20_ in striatum), we limited the analysis to mouse cortex and hippocampus where only STEP_61_ is expressed. Second, we performed parallel negative control co-IP experiments using STEP KO mouse brain extracts to eliminate non-specific binding proteins. Among the identified STEP_61_-binding proteins, 8% were ion channels, receptors and transporters (Won et al., 2019). They include several ionotropic glutamate receptors, but only one metabotropic glutamate receptor mGluR5 (Fig. S1). Interestingly, the related group I mGluR1 was not detected, indicating mGluR5 binding to STEP_61_ is likely not through the interaction of Fyn with STEP_61_.

We began our investigations by confirming STEP_61_ binding to mGluR5 using anti-HA co-IP of lysates from transfected HEK293T cells expressing HA-tagged STEP_61_ and myc-tagged mGluR5 (Fig. 1A). Importantly, we were able to immunoprecipitate endogenous mGluR5 and STEP_61_ in extracts from mouse brain and rat primary cortical neurons (Fig. 1B and C). By comparison, we found that little mGluR1 co-immunoprecipitated with STEP_61_ (Fig. 1C). In both mouse brain and dissociated neurons, the known STEP_61_ binding protein Fyn kinase was also present in the immunoprecipitates, whereas the negative control GAPDH was not.

Immunogold electron microscopy (EM) was employed to elucidate the localization of expressed GFP or myc tagged STEP_61_ and endogenous mGluR5 at excitatory synapses in dissociated rat hippocampal neurons. Additionally, the results of STEP_61_ were compared to those obtained with immunogold EM of EYFP-tagged PSD-95 (Fig. 1D–G). Measurements of the distance from the center of silver enhanced gold particles to the post-synaptic membrane showed two distinct pools of STEP_61_ at excitatory synapses (Fig. 1D). Near the extra-synaptic membrane outside the PSD, STEP_61_ located to the membrane, consistent with its role as a membrane protein. By contrast, membrane-particle distances were significantly larger in the subsynaptic zone located underneath the electron-dense PSD (Fig. 1E), suggesting that STEP_61_ might have different functions at the extra-synaptic membrane versus in the PSD (Won et al., 2019). To gain further insights, we compared STEP_61_ label in the PSD zone with that of PSD-95-EYFP which accumulated prominently in core PSD structure (Fig. 1D) (Chen et al., 2011). The mean distance of 27.3 ± 7.4 nm between PSD-95-EYFP label and the postsynaptic membrane was significantly smaller than that of STEP_61_ in PSD zone by 13 nm, suggesting that under our experimental conditions, this pool of STEP_61_ is not associated with the plasma membrane. STEP_61_ is known to interact with PSD-95 (Won et al., 2016), however in our experimental conditions this STEP_61_ is significantly further away from PSD-95 C-terminus. Earlier immunoEM mapping and EM tomography studies (Chen et al., 2008, 2011, 2015, 2018) have shown that at the PSD, PSD-95 appears as membrane associated vertical filaments with its N-terminus at the membrane and C-terminus away from the membrane in extended configuration, suggesting that perhaps the presence of the core PSD structure such as PSD-95 prevented STEP_61_ from reaching deeper into the core structure of the PSD. Alternatively, this pool of PSD zone STEP_61_ might not fully incorporated into the core PSD structure properly due to other interactions. At the extra-synaptic membrane, there are few PSD-95 labels, whereas there are extensive labels of STEP_61_ (Fig. 1E).

Immunogold EM of endogenous mGluR5 in hippocampal neurons showed labeling at peri-synaptic membrane locations outside the electron dense PSDs, whereas there was essentially no label within the PSD itself (Fig. 1F). To quantitively assess mGluR5 distribution, we measured the mean distance of mGluR5 immunogold label to the plasma membrane and found that it was not statistically different from the corresponding mean distance of peri-synaptically localized STEP_61_ (mGluR5: 13.0 ± 8.0 nm vs. STEP_61_: 11.4 ± 4.3 nm; Fig. 1G). Next, we determined the mean lateral distances of STEP_61_ and mGluR5 label to the outer edge of PSDs and found similar values for both (STEP_61_: 62.7 ± 56.4 nm vs. mGluR5: 69.1 ± 78.9 nm; Fig. 1G). In addition, our data showed that the vast majority (close to 80%) of both STEP_61_ and mGluR5 label is located within 100 nm from the PSD (Fig. 1G). Our immunogold EM results on mGluR5 in cultured rat hippocampal synapses agreed well with what was observed by an earlier immunoEM study of postsynaptic mGluR5 localization and membrane distribution in perfused rat brain hippocampal synapses (Lujan et al., 1996) and also agree quantitatively with the peri-synaptic distribution of mGluR5 in the post-synaptic membrane of cultured rat hippocampal synapses recently revealed by super resolution light microscopy (Scheefhals et al., 2023). Thus, our EM data demonstrated that STEP_61_ and mGluR5 co-localize in peri or extra-synaptic membranes in the proximity to the outer edge of the PSD.

### 3.2. Domain mapping of mGluR5-binding regions in STEP_61_

Using the AlphaFold protein structure prediction tool, we evaluated potential mGluR5 binding regions in STEP_61_. This prediction revealed possible interactions of the KIM and KIS domains of STEP_61_ with the last 120 amino acids of the mGluR5 C-tail (Fig. 2A). To experimentally test which regions of STEP_61_ are involved in binding to mGluR5, we generated a series of myc-tagged STEP_61_ deletion constructs (Fig. 2B) and tested them in co-IP experiments using lysates prepared from HEK293T cells co-transfected with HA-tagged mGluR5 (Fig. 2C). Deletion of the protein tyrosine phosphatase (PTP) domain did not affect binding to mGluR5 when compared to full-length STEP_61_ (Fig. 2D). By contrast, additional removal of the kinase-specificity sequence (KIS) domain reduced mGluR5 binding by 30.7%. Interestingly, further deletion of the kinase-interacting motif (KIM) domain reduced mGluR5 binding by 76.4%, suggesting that both the KIM and KIS domains are involved in STEP_61_-mGluR5 interactions, as predicted by AlphaFold (Fig. 2C and D). Unexpectedly, the naturally occurring STEP_46_ isoform, lacking the two proline-rich and two transmembrane regions present in the N-terminal half of STEP_61_, also showed reduced binding to the mGluR5 (43% of STEP_61_). Taken together, the KIS and KIM domains as well as N-terminus of STEP_61_ are important for the binding to mGluR5.

### 3.3. mGluR5 expression is decreased by STEP_61_ expression

To investigate possible functional relationships between STEP_61_ and mGluR5, we began by analyzing the effects of STEP_61_ on total and surface mGluR5 expression in transfected HEK293T cells using surface protein biotinylation. As shown in Fig. 3A, mGluR5 expression was reduced to 43.7% and surface expression was reduced to 75.9% in the presence of STEP_61_ when compared to controls in which STEP_61_ was omitted (Fig. 3A and B). These findings are consistent with immunocytochemistry experiments showing strongly reduced surface mGluR5 labeling in the presence of STEP_61_ in transfected HEK293T cells, whereas co-expressing Fyn kinase was without effect on mGluR5 (Fig. S2). We then tested STEP_61_ effects on mGluR5 expression in cultured hippocampal neurons co-transfected with HA-tagged STEP_61_ and myc-tagged mGluR5, assessing both surface and intracellular mGluR expression levels using sequential myc antibody labeling under non-permeabilizing conditions and following permeabilization. We found that STEP_61_ expression decreased mGluR5 surface expression to 67.4% of controls without co-expression of STEP_61_ (Fig. 3C and D). These results demonstrate that STEP_61_ reduces both total and surface expression of mGluR5 in neurons and heterologous cells.

### 3.4. STEP_61_ promotes mGluR5 degradation via a lysosomal pathway

Our finding that STEP_61_ decreases total and surface mGluR5 expression prompted us to investigate if STEP_61_ promotes mGluR5 degradation via lysosomal or proteasomal pathways. Interestingly, lysosomal inhibition by chloroquine (50 μM) and NH_4_Cl (20 mM), but not proteasomal inhibition by MG-132 (1 μM) in HEK293T cells co-expressing myc-mGluR5 and HA-STEP_61_, restored total mGluR5 protein expression to levels that were not different from cells expressing only mGluR5 (Fig. 4). This result indicates that the reduction of mGluR5 expression by STEP_61_ is associated with the lysosome-mediated degradation pathway.

### 3.5. The expression and the tyrosine phosphorylation of mGluR5 are increased in STEP KO mouse brain

Because mGluR5 is a highly tyrosine-phosphorylated protein in synaptic membrane (Orlando et al., 2002) and we found that STEP_61_ binds to mGluR5, we next investigated whether mGluR5 expression or its tyrosine phosphorylation is changed in STEP KO mouse brain. Previous studies showed that mGluR5 and STEP_61_ are enriched in synaptic plasma-membrane (SPM) (Scheefhals et al., 2023; Won et al., 2016). We purified SPM of WT and STEP KO mouse brains. Then, we checked the expression level of mGluR5 in SPM (Fig. 5A) and we also performed immunoprecipitation using pan-phospho-tyrosine 4G10 antibody for tyrosine-phosphorylated proteins, then immunoblotting for mGluR5 (Fig. 5A). Interestingly, both total expression and tyrosine-phosphorylation of mGluR5 at SPM in STEP KO mouse brain are increased up to 45.3% and 17.5%, respectively (Fig. 5B and C). These results support a model in which STEP_61_ binds to mGluR5, dephosphorylates the tyrosine residues, and regulates the expression of mGluR5 at synapses.

### 3.6. Effect of mGluR5 tyrosine-phosphorylation deficiency mutants on expression

There are four tyrosine residues (Y963, Y1060, Y1155, and Y1163) in the intracellular C-tail region of mGluR5, and previous studies suggested that one or more tyrosine residues in the C-tail can be phosphorylated by Src family kinases (SFKs) such as Fyn (Amata et al., 2014; Mao and Wang, 2016; Orlando et al., 2002). Tyrosine residue Y821, embedded in last transmembrane domain, is useful as a negative control because it is known that STEP_61_ only dephosphorylates the cytosolic tyrosines on the target proteins (Nguyen et al., 2002; Snyder et al., 2005; Won et al., 2019; Xu et al., 2012). To examine which tyrosine residue is important for mGluR5 surface expression, we generated single (Y963A or Y1060A or Y1155A or Y1163A, plus Y821A as a negative control) and quadruple phosphorylation-deficient mutants (4 YAs: Y963A-Y1060A-Y1155A-Y1163A) (Fig. 6A). We then transfected hippocampal neurons with myc-tagged mGluR5-WT or phosphor-mutant mGluR5 variants and performed surface versus intracellular mGluR5 immunostaining using an anti-myc antibody as described above. As expected, surface expression of mGluR5-Y821A mutant was not different from WT. Interestingly, all four single Y to A mutations significantly reduced mGluR5 surface expression to 70.9% (Y693A), 57.5% (Y1060A), 48.7% (Y1155A), and 51.8% (Y1163A), respectively (Fig. 6B and C). Next, we tested mGluR5–4YAs and found that its surface expression was reduced to a similar degree (54.4% of WT control) compared to three of the four single Y to A mutants, indicating that there was no combinational effect of targeting multiple tyrosine residues. Fyn co-expression was unable to restore normal surface expression of mGluR5–4YAs. Conversely, STEP_61_ co-expression did not further decrease mGluR5–4YAs surface expression, consistent with the idea that the Y to A mutations occluded the effect of STEP_61_-mediated tyrosine dephosphorylation on mGluR5 surface expression (Fig. 6D and E). In a parallel set of experiments, we were able to confirm these results in HEK293T cells (Fig. S3). The tyrosine deficient mutant, 4 YAs, manifested a dramatic reduction surface expression that was not responsive to the co-expressed Fyn and STEP_61_. When we co-transfected hippocampal neurons with mGluR5 and STEP_61_, the surface expression of mGluR5 was significantly decreased (Fig. 3). Next, we tested mGluR5–4YAs phosphor-tyrosine levels by co-transfecting myc-tagged mGluR5-WT or 4 YAs mutant with Fyn in HEK293T cells, followed by IP using the pan-phospho-tyrosine antibody 4G10 and immunoblotting with mGluR5 (Fig. 6F). Interestingly, mGluR5–4YAs expression was reduced to 26.9% and its tyrosine phosphorylation level was also decreased to 56% (Fig. 6G). Altogether, these results demonstrated the role of STEP_61_ regulating mGluR5 expression in a tyrosine-dephosphorylation-dependent manner.

## 4. Discussion

mGluR receptors are G protein-coupled receptors that bind to glutamate to regulate synaptic plasticity, neuronal excitability, and intracellular signaling (Niswender and Conn, 2010; Ribeiro et al., 2010; Nakanishi, 1994; Suh et al., 2018; Vaidya et al., 2013). Group I receptors are coupled to Gq/_11_-like proteins and stimulate phospholipase C (PLC), leading to an increase of intracellular Ca^2+^ and activation of PKC. mGluR5 belongs to the group I mGluRs, which possess a large extracellular N-terminal domain and a long intracellular C-terminal tail. The long cytosolic C-tail plays important roles in regulating mGluR5 trafficking events such as endocytosis. In addition, posttranslational modification such as phosphorylation target the C-tail and provide dynamic and compartment-specific regulation. (Ribeiro et al., 2010). For example, mGluR5 desensitization is regulated by the activity of a variety of serine/threonine protein kinases including PKC, PKA, Ca^2+^/calmodulin-dependent protein kinase II (CaMKII), and MAPKs (Choi et al., 2011a,b; Kim et al., 2008; Lee et al., 2008). However, less is known about the regulation of mGluR5 trafficking and function by its tyrosine phosphorylation.

STEP is a brain-specific protein tyrosine phosphatase, which has splice variants including STEP_61_ and STEP_46_. STEP_61_ is the longest form which contains two transmembrane domains, two proline-rich domains in the N-terminus and KIM and KIS domains, substrate binding regions, in the middle, and PTP domain showing protein tyrosine phosphatase activity in the C-terminus. Due to two transmembrane domains in the N-terminus, STEP_61_ is known to be an integral membrane protein that is expressed at cell surface, whereas STEP_46_ is a cytosolic protein. Notably, all STEP splice variants are expressed in the striatum, whereas only STEP_61_ is expressed in cortex and hippocampus (Won et al., 2019). We have previously reported that STEP_61_ binds to the NMDA receptor subunit GluN2B and the AMPA receptor subunit GluA2, and STEP_61_ regulates differentially NMDAR and AMPAR trafficking by dephosphorylating a tyrosine residue in their C-tail (Won and Roche, 2021).

Tyrosine phosphorylation regulates both iGluRs and mGluRs protein interactions, trafficking, and function (Lavezzari et al., 2004; Mao and Wang, 2016; Wang and Salter, 1994). Src family kinases such as Fyn and Src are expressed throughout the brain (Amata et al., 2014; Mao and Wang, 2016) and are important for the regulation of receptors and synapses under normal and pathological conditions in neurons (Amata et al., 2014). Our previous work showed that STEP_61_ binds to Fyn, and Fyn has been reported to bind to mGluR1 and to phosphorylate a tyrosine residue (Y937) within its intracellular C-tail (Jin et al., 2017). Similarly, mGluR5 has been shown to be heavily tyrosine phosphorylated (Orlando et al., 2002) in rodent brain; however, no non-receptor kinases have been demonstrated to either directly interact with or phosphorylate mGluR5. There are, however, reports of an association between STEP and mGluR5, including one paper that showed mGluR5 activation using (S)-3,5-dihydroxyphenylglycine (DHPG) increased STEP translation in mitogen-activated protein kinases and phosphoinositide-3 kinase pathways and promoted STEP_61_ mediated endocytosis of AMPA receptors (Zhang et al., 2008). Similarly, an interaction between Adenosine A_2A_ receptors and mGluR5 mediates STEP activation and promotes STEP/mGluR5 binding in mouse brain (Mallozzi et al., 2023). Both Fyn and ERK1/2 bind and are substrates for STEP_61_, and Fyn-phosphorylated mGluR5 activates ERK1/2 downstream. Our findings now show that both Fyn and STEP_61_ can bind to mGluR5, to phosphorylate or dephosphorylate tyrosine residues in C-tail of mGluR5, revealing a critical mechanism for regulating mGluR5 activation, trafficking and downstream signaling.

Using an in-gel mass spectrophotometry method, we previously characterized the STEP_61_ interactome (Won et al., 2019), which included 26 ion channels, receptors, and transporters among a total of 315 binding proteins. Interestingly, there were not only known iGluRs (i.g. GluN2A, GluN2B, GluN1, and GluA2) identified, but also mGluRs such as mGluR5 and mGluR3 on the list of identified STEP_61_ interactors. Although, there are previous studies about the association between STEP_61_ and mGluR5 (Mallozzi et al., 2023; Zhang et al., 2008), little is known about the details of the direct binding and the downstream consequences. In this paper, we showed that STEP_61_ binds to mGluR5 in various tissues including mouse brain, cultured primary neurons, and heterologous cells expressing mGluR5. Importantly, we also showed that STEP_61,_ like mGluR5, is enriched in extra-synaptic region of neurons with identical spatial distribution using immune EM (Fig. 1). We also performed domain mapping of STEP_61_ to determine the key binding regions required for binding to mGluR5. We found that the KIM domain of STEP_61_ is the major binding domain, but it requires the N-terminus of STEP_61_ to enhance binding to mGluR5. The first 120 amino acid of the proximal C-tail of mGluR5 constitutes a binding region for STEP_61_. We found that STEP_61_ regulates mGluR5 levels through lysosomal degradation pathway as previous studies showed internalized mGluR5 can be degraded in lysosomes (Ko et al., 2012; Toledano-Zaragoza et al., 2024). All four tyrosine residues in mGluR5 C-tail can serve as substrates for STEP_61_, which can remarkably inhibit surface trafficking of mGluR5 in both neurons and HEK293T cells. We also demonstrated that a brain-enriched nRTKs, Fyn, phosphorylates tyrosine residues in mGluR5 C-tail. However, Fyn overexpression cannot elevate the surface expression of mGluR5 in neurons or in HEK293T cells, suggesting that overexpressed mGluR5 may be already activated and saturated for surface trafficking by endogenous Fyn and/or Src.

Glutamate binds to mGluR5 and leads to receptor activation, thereby propagating downstream signaling. Also, glutamate treatment results in dephosphorylation and subsequent activation of STEP_61_. This activated STEP_61_ dephosphorylates its target proteins such as p38 MAPK (Rajagopal et al., 2021). These findings suggest that STEP_61_ is activated by glutamate and binds to the C-tail of mGluR5 and dephosphorylates tyrosine residues in its C-tail, regulating mGluR5 trafficking. On the other hand, we confirmed mGluR5 binding with STEP_61_ in heterologous cells, cultured neurons, and brain tissues without glutamate treatment. These results indicate that mGluR5 activation by glutamate is not necessary for its binding with STEP_61_. In this paper, we showed the KIM and KIS domains located in the middle of STEP_61_ are important for the binding to the mGluR5 C-tail. As STEP_61_ is a transmembrane protein with two membrane-spanning regions in its N-terminus (Won et al., 2016), the binding of STEP_61_ to mGluR5 might affect mGluR5 binding proteins, particularly membrane-associated proteins such as G-protein complexes. Therefore, this STEP_61_-mGluR5 binding may interfere with the coupling of mGluR5 to heterotrimeric G proteins as G protein-coupled receptor kinase 2 (GRK2) uncouples mGluR5 from heterotrimeric G protein complex through promoting the binding of β-arrestin proteins (Ribeiro et al., 2009). As we have shown, STEP_61_ dephosphorylates tyrosine residues in the mGluR5 C-tail and regulates mGluR5 expression via the lysosomal degradation pathway. Our data suggest a model in which dephosphorylation of mGluR5 by STEP_61_ no longer exerts downstream signaling via PLCβ because dephosphorylated mGluR5 is endocytosed and degraded in a lysosomal-dependent manner. The function of STEP_61_ regulation of mGluR5 at the cell surface may or may not be influenced by mGluR5 negative allosteric modulators (NAMs), such as MPEP, as these compounds interact with the mGluR5 transmembrane regions (Li et al., 2024). However, our data are consistent with mGluR5 regulation by STEP_61_ being independent of functional regulation by mGluR5 NAMs because STEP_61_ regulates mGluR5 trafficking via dephosphorylation of its C-tail. Taken together, our report demonstrated a direct interaction of mGluR5 with STEP_61_, and STEP_61_ negatively regulates the expression of mGluR5 via dephosphorylating its C-tail in primary cultured neurons and *in vivo*.

## Supplementary Material

3

4

5

6

7

## Figures and Tables

**Fig. 1.**
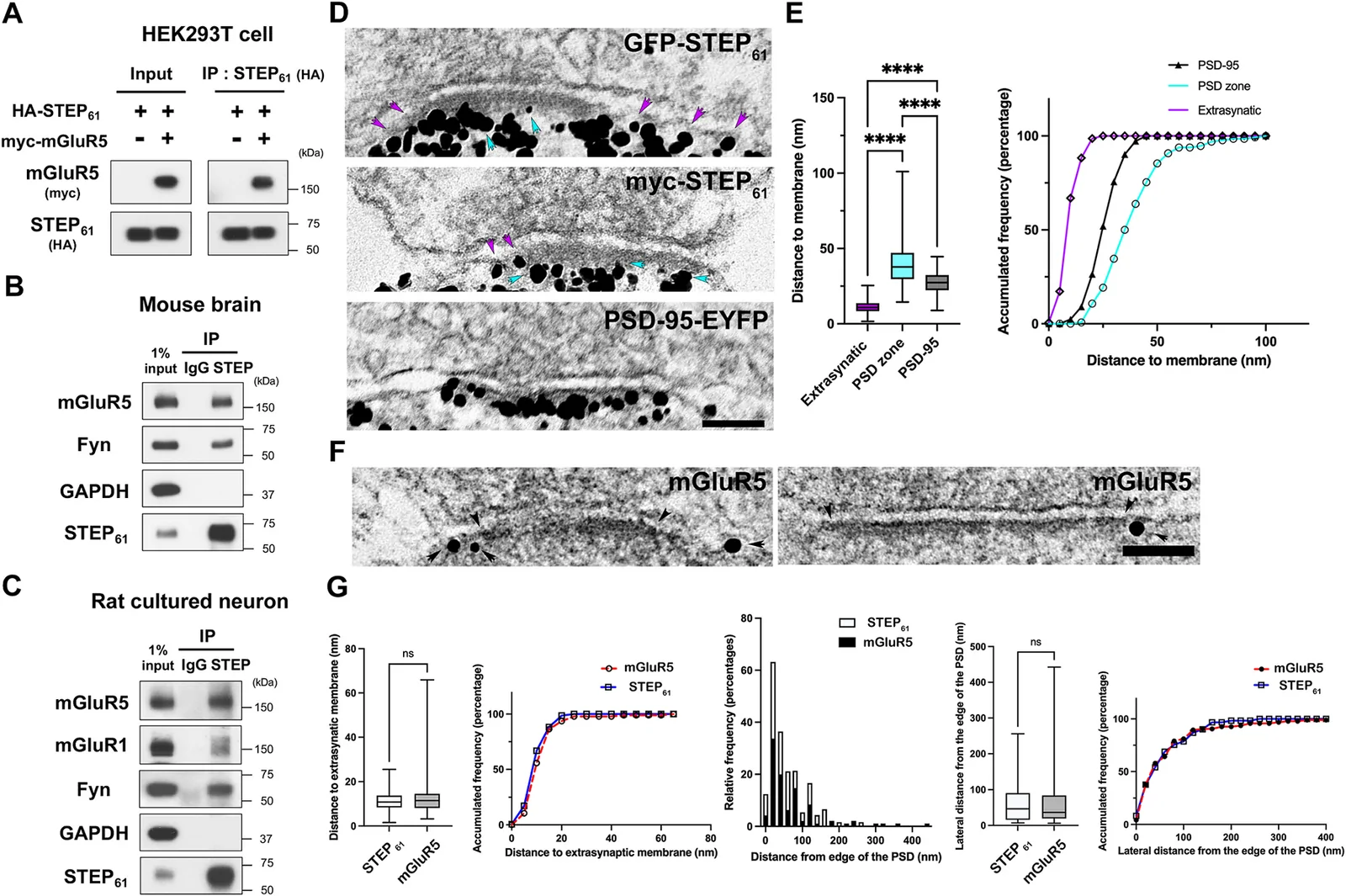
STEP_61_ localizes to extra-synaptic sites and binds to mGluR5. A. HEK293T cells were transfected with HA-STEP_61_ and myc-mGluR5 and solubilized with RIPA buffer. IP was performed with a HA antibody for STEP_61_, and then immunoblotted with myc antibody for mGluR5. B-C. P2 fractions of adult mouse brain (B) and primary rat cortical neurons at *DIV* 28 (C) were solubilized with 1% sodium deoxycholate (Doc) lysis buffer, neutralized with 1% Triton X-100 lysis buffer, and immunoprecipitated (IP) with STEP antibody or IgG as control, and then immunoblotted with indicated antibodies. D. Immunogold electron microscopy (EM) localization of STEP_61_ and mGluR5 in rat hippocampal synapses in comparison to PSD-95. Two distinctive pools of STEP_61_ at extra-synaptic membrane and the PSD zone (top and middle left images): immunogold labels of GFP or myc tagged and transiently expressed STEP_61_ at the extra-synaptic membrane (purple arrows) and in the PSD zone underneath the core electron dense PSD (cyan arrows) showing significantly different distances to the postsynaptic membrane. (Third left). Transmission EM image of immunogold labels of overexpressed C-terminal-tagged PSD-95-EYFP that are concentrated at the core layer of the PSD and distribute prevalently across the PSD. Scale bar, 100 nm. E. Distance measurements of silver enhanced gold particles (center) to the post-synaptic membrane reveals two populations of STEP_61_ at synapses. Data (GFP-STEP_61_ and myc-STEP_61_ measurements combined) shown as summary box whisker plot of distance measurements for STEP_61_ (left): STEP_61_ (Extra-synaptic membrane) 11.4 ± 4.3 nm, (mean ± SD), min = 1.6 nm, max = 25.5 nm, median = 10.9 nm, N = 145 (total number of gold particles pooled from 12 synapses); STEP_61_ (PSD zone): 40.0 ± 15.4 nm, min = 14.5 nm, max = 101.1 nm, median = 37.8 nm, N = 130 (total number of gold particles pooled from 12 synapses); C-terminal EYFP tagged PSD-95: 27.3 ± 7.4 nm, min = 8.9 nm, max = 44.6 nm, median = 27.4 nm, N = 254 (total number of gold particles from 19 synapses). ****p < 0.0001 (one way-ANOVA, Tukey’s multiple comparison test). Cumulative distribution of immunogold label distances to postsynaptic membrane for STEP_61_ at the extra-synaptic membrane and PSD zone versus PSD-95 in the PSD (right). F. Immunogold labels of endogenous mGluR5 are localized outside the PSD at the extra-synaptic membrane. Black arrows: mGluR5 labels; black arrowhead: edge of the electron dense PSD. Scale bar, 100 nm. G. At the extra-synaptic membrane, STEP_61_ and mGluR5 immunogold label distances to the post-synaptic membrane distributions are similar. mGluR5: 13.0 ± 8.0 nm, min = 3.2 nm, max = 65.9 nm, median = 11.5 nm, N = 95 (total number of gold particles from 47 synapses). STEP_61_: 11.4 ± 4.3 nm, min = 1.6 nm, max = 25.5 nm, median = 10.9 nm, N = 145 (total number of gold particles from 12 synapses). Unpaired *t-test*, Mann-Whitney test, *p =* 0.2019 (not significant). Data shown as box whisker plot (left) and cumulative distribution plots (right). At the extra-synaptic membrane, STEP_61_ and mGluR5 immunogold labels showed similar lateral distance from the edge of PSDs. mGluR5: 69.1 ± 78.9 nm, min = 6.0 nm, max = 442.6 nm, median = 36.4 nm, N = 94 (total number of gold particles from 47 synapses); STEP_61_: 62.7 ± 56.4 nm, min = 7.1 nm, max = 255.8 nm, median = 46.3 nm, N = 61(total number of gold particles pooled from 12 synapses). Unpaired *t-test*, Mann-Whitney test, *p =* 0.8245, not significant. Histogram (left), box whisker plot (middle), and accumulated lateral distance distribution (right). Number of independent immunogold EM experiments: 2 (PSD-95-EYFP), 2 (GFP-STEP_61_), 1 (myc-STEP_61_), 2 (mGluR5).

**Fig. 2.**
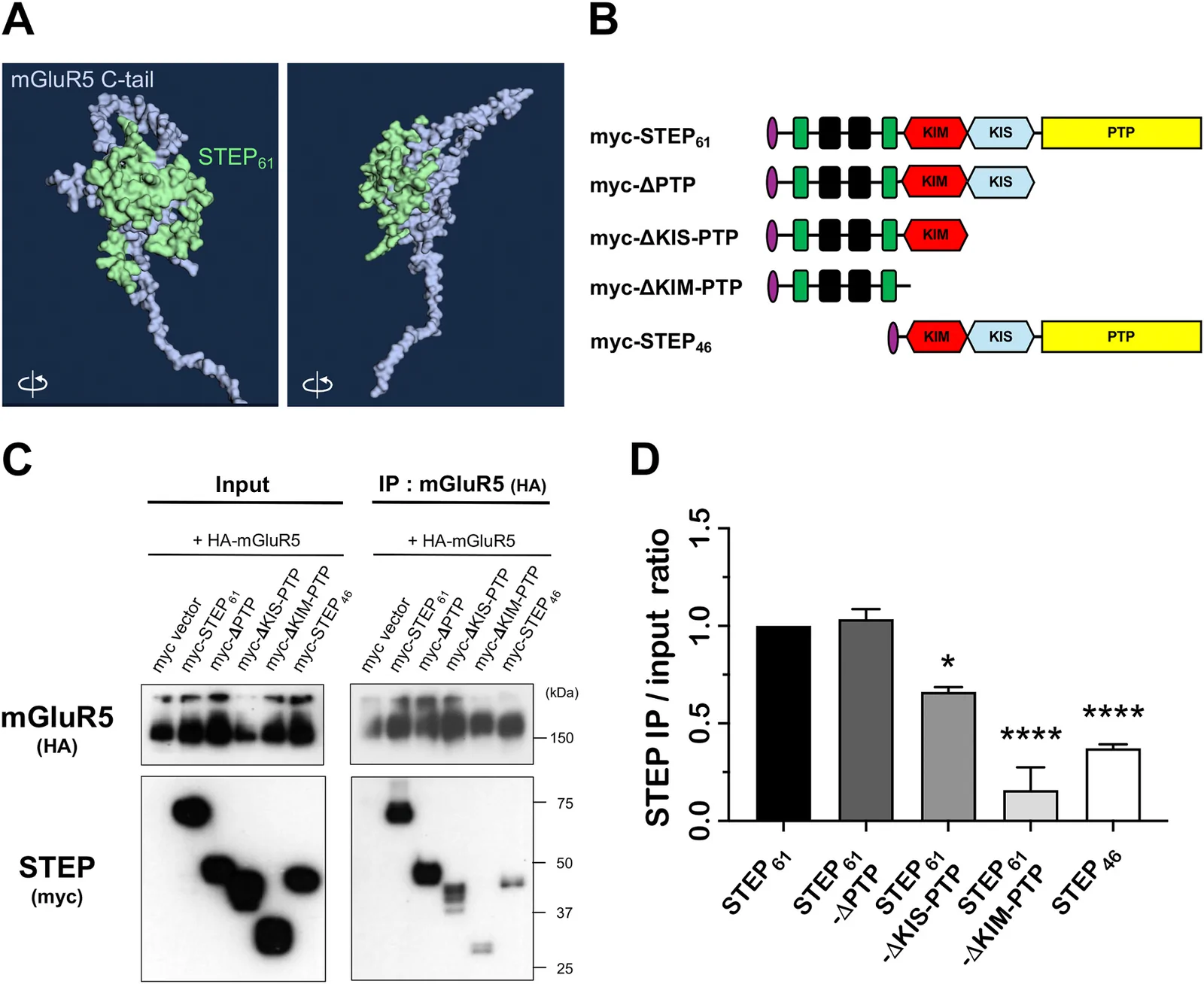
The N-terminus, KIM, and KIS domains of STEP_61_ are important for mGluR5 binding. A. AlphaFold2-predicted, structural interactions between the last 120 amino acids of the mGluR5 C-tail (light blue) and the KIM and KIS domains of STEP_61_ (green), shown in front (left) and lateral views (right). B. Schematic representation of the domain structure of STEP_61_ full-length and truncation constructs: myc tag (purple), proline-rich domain (green), transmembrane domain (black), KIM domain (red), KIS domain (light blue), and PTP domain (yellow). C. Representative co-immunoprecipitation Western blot of lysates from HEK293T co-transfected with HA-tagged mGluR5 and myc-tagged STEP constructs. D. Densitometric analysis of immunoprecipitated STEP_61_ band intensities. Data are normalized to full-length STEP_61_ and represent the mean + S.E.M. of three independent experiments. ***p < 0.001.

**Fig. 3.**
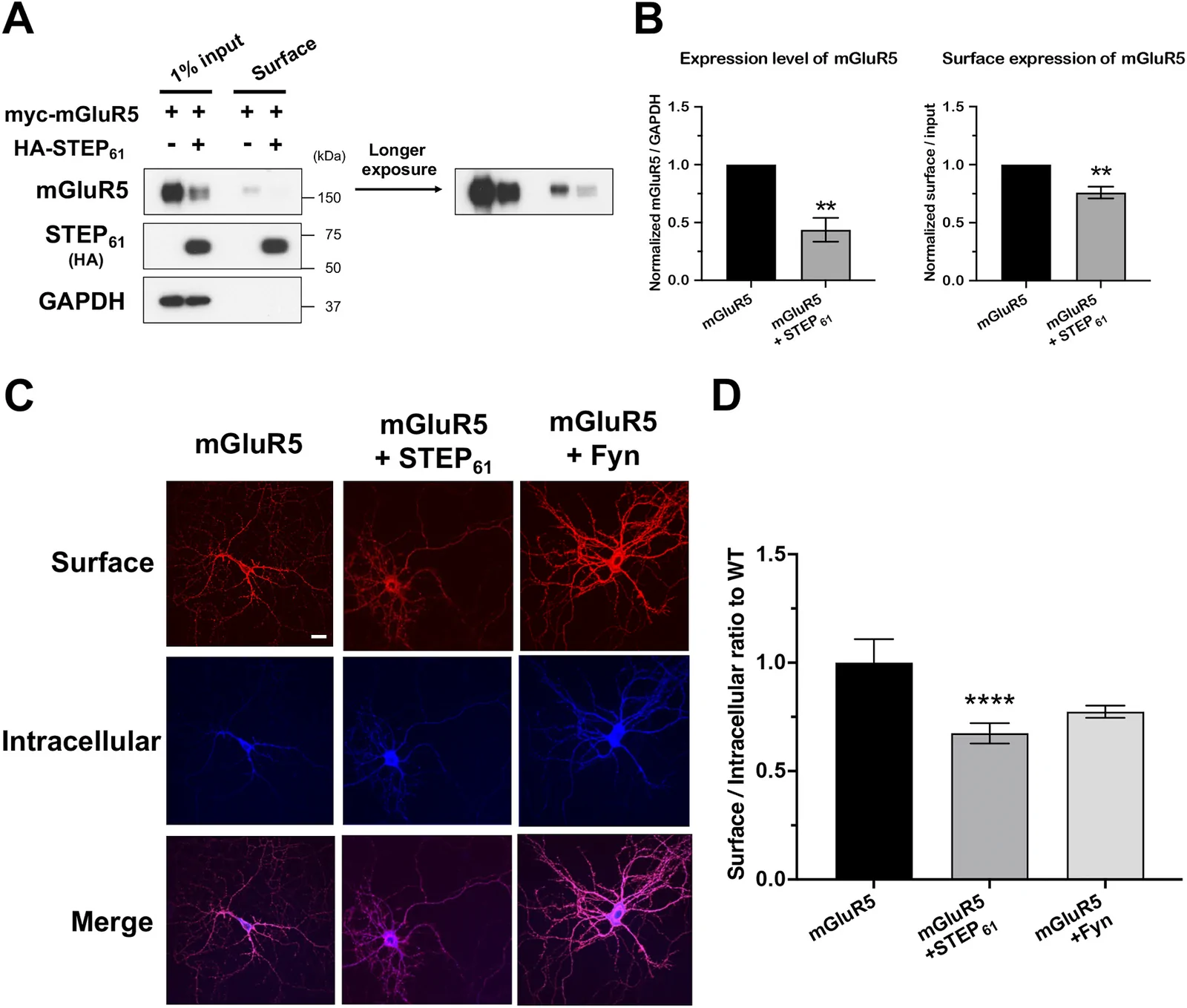
STEP_61_ regulates the surface expression of mGluR5 in heterologous cells and neurons. A. Representative surface biotinylation assay in HEK293T cells co-transfected with myc-tagged mGluR5 and HA-tagged STEP_61_, performed one day after transfection as described in Materials and methods. B. Densitometric analysis of Western blot band intensities. Total mGluR5 protein levels are normalized to GAPDH, surface mGluR5 levels are plotted as surface/input ratios, normalized to control experiments in which mGluR5 was expressed alone. Data represent the mean ± S.E.M. of three independent experiments. **p < 0.01. C. Representative confocal images of primary hippocampal neurons transfected at *DIV* 10–11 with myc-mGluR5, HA-STEP_61_, and Fyn and then fixed at *DIV* 12–13 for sequential surface and intracellular mGluR5. Scale bar, 20 μm. D. Images were analyzed for surface and intracellular mGluR5 immunostaining using ImageJ. Data are plotted as surface:intracellular ratios, normalized to control experiments omitting STEP_61_ and represent the mean ± S.E.M. of five independent experiments. ****p < 0.001 (one-way ANOVA with Dunnett’s post hoc test). Scale bar, 20 μm.

**Fig. 4.**
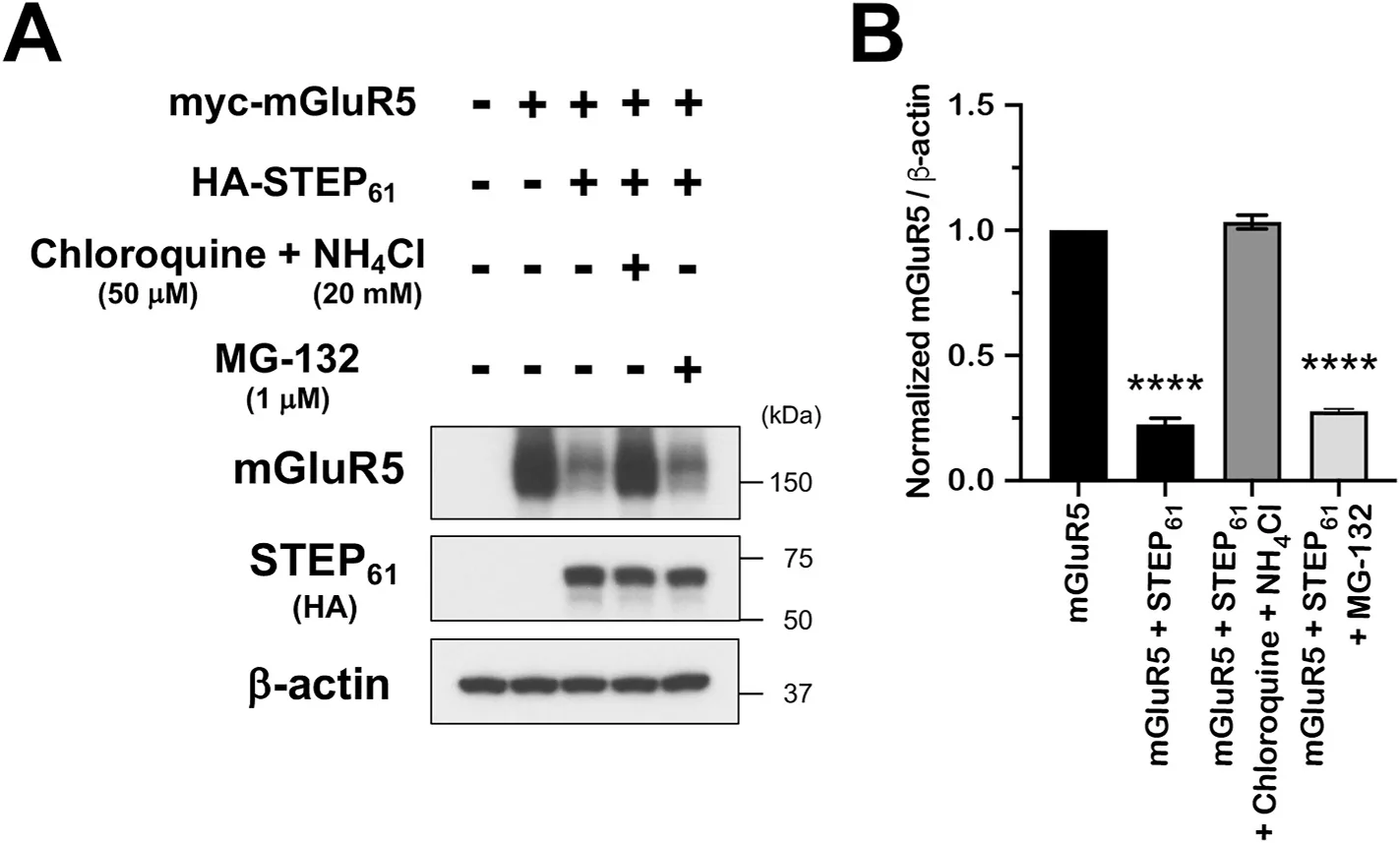
STEP_61_ regulates mGluR5 expression through lysosomal degradation. A-B. HEK293T cells were transfected with myc-tagged mGluR5 and HA-tagged STEP_61_. The next day after transfection, cells were treated with 50 μM chloroquine and 20 mM NH_4_Cl or 1 μM MG-132 for 6 h. Cells were solubilized with RIPA buffer, then immunoblotted with indicated antibodies. Three independent experiments were performed. A band intensity was measured using ImageJ, and mGluR5 was divided by β-actin, then normalized with mGluR5 expression alone. Error bars represent ± S.E.M. Dunnett’s one-way ANOVA test was performed for statistical analysis. ****p < 0.001.

**Fig. 5.**
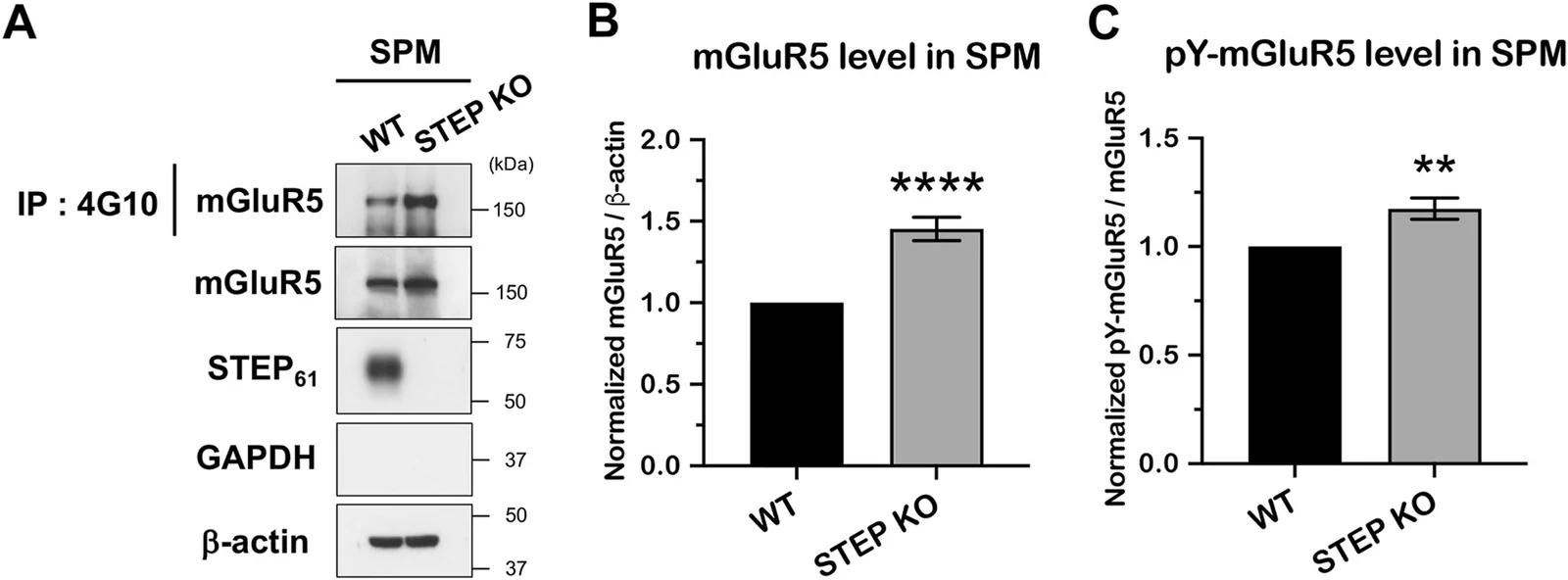
mGluR5 protein levels and tyrosine phosphorylation are increased in STEP KO mouse brain. A-C. Synaptic plasma-membranes (SPMs) were isolated from adult WT and adult STEP KO mouse brains. SPMs were solubilized with 1% SDS lysis buffer and neutralized with 1% Triton X-100 lysis buffer, and immunoprecipitated with 4G10 antibody (pan phospho-tyrosine antibody) as described in Materials and methods, then immunoblotted with mGluR5 antibody. Four independent experiments were performed. A band intensity was measured using ImageJ. For total mGluR5 and tyrosine-phosphorylated (pY) mGluR5, mGluR5 was divided by β-actin and pY-mGluR5 was divided by mGluR5, then normalized with WT. Error bars represent ± S.E.M. Student’s *t-test* was performed for statistical analysis. **p < 0.01, ****p < 0.001.

**Fig. 6.**
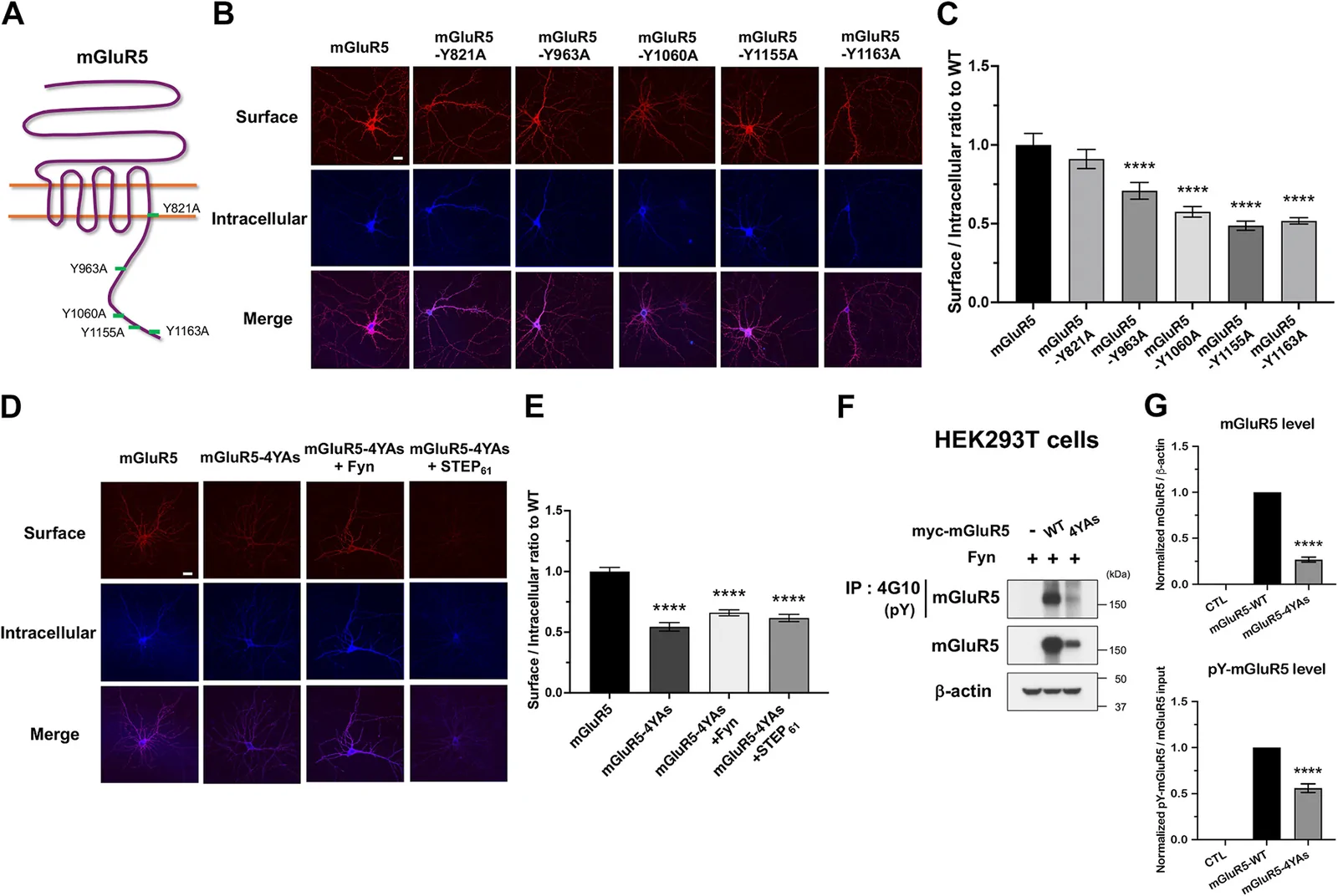
The surface expression of mGluR5 Y-A mutants is decreased in neurons. A. Schematic depiction of targeted tyrosine residues in the C-tail of mGluR5 (membrane-embedded Y821A included as negative control). B. Representative confocal images of cultured hippocampal neurons transfected at *DIV* 10–11 with mGluR5-WT, -Y821A, -Y963A, -Y1060A, -Y1155A, and -Y1163A. Neurons were fixed at *DIV* 12–13 and sequentially labeled for surface and intracellular mGluR5 using an anti-myc antibody. Scale bar, 20 μm. C. Summary analysis of the effects of Y to A mutations in the C-tail on mGluR5 surface expression. Data plotted as in Fig. 3 and represent the mean ± S.E.M. of five independent experiments. ***p < 0.001 (One-way ANOVA with Dunnett’s post hoc test). D-E. Representative confocal images and quantitative analysis of cultured hippocampal neurons transfected at *DIV* 10–11 with mGluR5-WT or −4YAs (Y963A, Y1060A, Y1155A, and Y1163A) constructs and Fyn or STEP_61_ constructs as indicated. Experiment was performed and analyzed as described in panels B and C. Scale bar, 20 μm. F. Analysis of phospho-tyrosine levels in HEK293T cells transfected with Fyn kinase and myc-tagged mGluR5-WT or −4YAs. Phospho-tyrosine IP was performed one day after transfection and the Western blot was probed for mGluR5. A negative control was included to confirm the specificity of the mGluR5 signal following anti-phospho-tyrosine IP. G. Densitometric analysis of representative Western blotting results in F. The graph in G (top) shows β-actin normalized mGluR5 protein levels, relative to WT. Similarly, the graph G (bottom) shows phospho-tyrosine mGluR5 band intensities normalized to the corresponding total mGluR5 band intensities, relative to WT. Data in G represent the mean ± S.E.M. of three independent experiments. Student’s *t-test* was performed for statistical analysis. ****p < 0.001.

## Data Availability

The data that support the findings of this study are available from the corresponding author upon reasonable request.

## Appendix A. Supplementary data

Supplementary data to this article can be found online at https://doi.org/10.1016/j.neuropharm.2026.111198.
