# Supplementary material for: STEP61 is a regulator of mGluR5 expression
Source: Neuropharmacology. Author manuscript; Available in PMC 2026 Oct 27. (PMC13712166; doi:10.1016/j.neuropharm.2026.111198)
Supplement: Supplementary file 1 [file NIHMS2214490-supplement-7.docx]

**Supplementary material**

**LC/MS/MS sample treatment and Data Analysis**

Detailed methods for the in-gel affinity-purification-LC/MS/MS including sample treatment, procedures and data analysis have been published (Won et al., *PNAS*, 2019).

**Supplementary Fig. 1. Identification of mGluR5 on the list of STEP_61_-interacting proteins.**

From the previous study (Won et al., *PNAS*, 2019), a MS/MS spectrum of a peptide, _115_DSLISSEEEEGLVR_128_ of mGluR5 amino acid sequence was identified.

**Supplementary Fig. 2. STEP_61_ expression reduces mGluR5 surface expression in HEK293T cells.**

A-B. HEK293T cells were transfected with myc-mGluR5, Fyn, and HA-STEP_61_. One day after transfection, immunostaining for surface and intracellular mGluR5 was performed, and images were captured using a confocal microscope and then analyzed for surface and intracellular mGluR5 immunostaining using ImageJ. N=5. ANOVA test was performed for statistical analysis. *** *p* < 0.001. Scale bar, 20 μm.

**Supplementary Fig. 3. The surface expression of mGluR5-4YAs mutant is decreased in HEK293T cells.**

A-B. HEK293T cells were transfected with mGluR5-WT, mGluR5-4YAs, Fyn, and STEP_61_ and then immunostained for surface and intracellular mGluR5. Images were analyzed using ImageJ and ANOVA test was performed for statistical analysis. N=5. *** *p* < 0.001. Scale bar, 20 μm.

**Supplementary Fig. 4. The immunoblots in main figures.**

As the protein sizes are different, the PDVF membrane after protein gel transferred was cut based on each protein size, then blot was applied using primary and secondary antibodies.
